# Virtual Environments for Visualizing Structural Health Monitoring Sensor Networks, Data, and Metadata

**DOI:** 10.3390/s18010243

**Published:** 2018-01-16

**Authors:** Rebecca Napolitano, Anna Blyth, Branko Glisic

**Affiliations:** Civil and Environmental Engineering Department, Princeton University, Princeton, NJ 08544, USA; ablyth@princeton.edu (A.B.); bglisic@princeton.edu (B.G.)

**Keywords:** visualization, structural health monitoring, sensor networks, data, metadata

## Abstract

Visualization of sensor networks, data, and metadata is becoming one of the most pivotal aspects of the structural health monitoring (SHM) process. Without the ability to communicate efficiently and effectively between disparate groups working on a project, an SHM system can be underused, misunderstood, or even abandoned. For this reason, this work seeks to evaluate visualization techniques in the field, identify flaws in current practices, and devise a new method for visualizing and accessing SHM data and metadata in 3D. More precisely, the work presented here reflects a method and digital workflow for integrating SHM sensor networks, data, and metadata into a virtual reality environment by combining spherical imaging and informational modeling. Both intuitive and interactive, this method fosters communication on a project enabling diverse practitioners of SHM to efficiently consult and use the sensor networks, data, and metadata. The method is presented through its implementation on a case study, Streicker Bridge at Princeton University campus. To illustrate the efficiency of the new method, the time and data file size were compared to other potential methods used for visualizing and accessing SHM sensor networks, data, and metadata in 3D. Additionally, feedback from civil engineering students familiar with SHM is used for validation. Recommendations on how different groups working together on an SHM project can create SHM virtual environment and convey data to proper audiences, are also included.

## 1. Introduction

Structural Health Monitoring (SHM) is a process that provides accurate and in-time information about a structure’s condition and performance [1]. SHM can be broken down into five core monitoring activities: (1) defining of SHM strategy; (2) installation of SHM system; (3) maintenance of SHM system; (4) data and metadata management; (5) closing activities (if any) [1]. In this work, data and metadata management are discussed, in particular, visualization and accessibility, which are crucial components to the success of an SHM system. The term “data” refers to measured and recorded parameters of the structure using an SHM system (e.g., time series of strain, acceleration, etc.), as well as results of their analysis (e.g., deformed shape, natural frequency, evaluation of prestress force, etc.). Term ”metadata” refers to data describing the structure and the SHM system (e.g., plans with locations of sensors, type and specification of sensors, etc.). To simplify narration in the further text, the words “metadata” and “accessibility” will be omitted, but should be considered as included whenever the term “data visualization” is mentioned, unless specified differently.

Visualization of data and sensor networks between the parties involved in SHM, i.e., users of SHM (owners, consultants, technology providers, contractors, researchers, etc.) is commonly a “bottleneck” for SHM that can lead to inefficient utilization of SHM or even complete abandonment of the system [1]. The sensor network and data associated with an SHM project need to be easily accessible, understandable, and transferrable. The main principles of visualization for SHM are presented in previous work [2]. The research presented here identifies the needs for new typology of sensor network and data visualization in 3D, creates a new method that addresses the identified need, and evaluates the method through real-life application, Streicker Bridge on Princeton University’s campus. The method integrates visualization of sensor networks and data in a virtual environment, where the latter consists of Virtual Tour (VT) and Informational Modelling (IM). Evaluation includes a short survey carried-out among graduate and undergraduate students.

## 2. Current Methodologies for Documentation and Communication

In general, there are two main typologies for visualizing sensor networks and data in SHM: two-dimensional (2D) and three-dimensional (3D). 2D methods for documenting the locations of SHM sensors on a structure include Computer-Aided Design (CAD) programs. These enable a user to create scaled drawings of a structure and label the locations of sensors. Additionally, there are other 2D programs that enable a user to visualize data with still images from a structure. For example, many SHM systems have their own proprietary 2D visualization software [3,4,5,6]. While 2D methods can be valuable for a project since they are less costly in terms of time, money, and data management than their 3D counterparts, these methods do not always capture the full details. Furthermore, multiple 2D graphics do not guarantee a comprehensive or intuitive understanding of a structure, sensor network, or SHM data and results. By conveying 3D objects (e.g., network of sensors or location and extent of damage) in 2D space, there can be miscommunication and misunderstanding between various SHM users with diverse backgrounds and interests. Furthermore, the future development and implementation of advanced sensors (e.g., 2D sensing sheets, skins, surfaces, drone-taken images, etc.) will make it more complicated to use 2D methods [7,8,9,10,11,12,13,14,15,16].

3D methods could be more advantageous for depicting complicated topologies of structures and networks of sensors in 3D space [17]. 3D methods for documenting an SHM project include technologies such as photogrammetry, laser scanning, and Building Information Modeling (BIM). Photogrammetry is the method of stitching together 2D images to create a scalable 3D point cloud; laser scanning creates a scalable 3D point cloud by calculating the distance a laser beam travels to every point on a structure; BIM enables 3D models to have interactive links with relevant information [18,19]. Bosche et al., (2009) used automated recognition of 3D objects in laser scan data to monitor a structure and site during the construciton process [20]. Virtual reality BIM models have also been used by many major companies including LERA and AECOM to facilitate the understanding of a complex space [21]. However, since very few structures today have as-built records or information models, Brilakis et al., (2010) outlined a novel method for generating BIM models based on video data and laser scan models [22]. David Mascarenas at Los Alamos National Laboratory’s National Security Education Center has developed both virtual reality and augmented reality environments for specifically structural health monitoring [23,24]. Currently, David Lattanzi at George Mason University is working to create a method where a BIM model can be overlaid with LiDAR images of a structure over the course of its life cycle [17]. Through these methods and others that are similar, diverse SHM users can communicate the relationship between different regions or objects of interest of a structure in a way that cannot be efficiently done by 2D methods. Yet, while these methods are well-suited for depicting 3D concepts about a structure, these 3D methods can be costly for a project in terms of time, money, and management.

**Figure 1:**
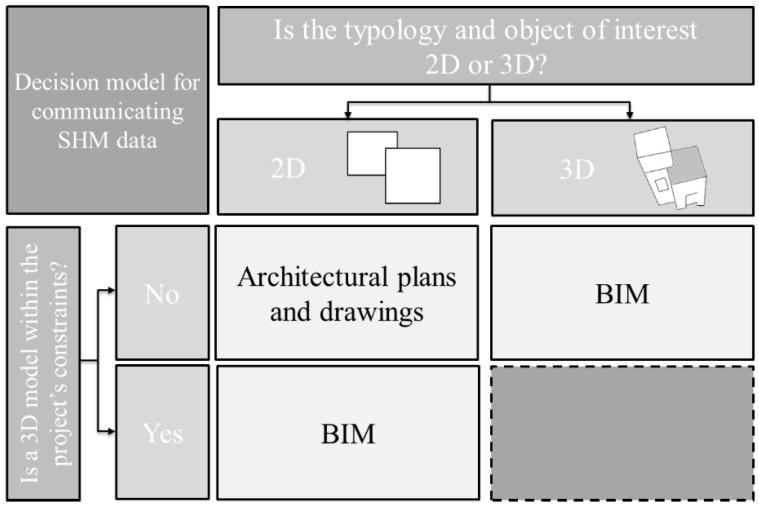
Decision model for documenting and communicating SHM data and metadata (Building images adapted from [26].

Thus, there is dichotomy in the presented 2D and 3D methods when used for SHM. Currently, efficient methods are available if (1) a 3D model is within a broader project’s scope and constraints (e.g., created for design or construction purposes, i.e., not built and used only for SHM purposes) [25] or (2) if the objects of interest (e.g., damage and sensor network) are simple enough so they can be represented in 2D. However, there are no methods that effectively communicate 3D topologies and objects of interests on an SHM project if a 3D digital model is not required within a broader project’s scope and constraints. Figure 1 illustrates this gap in current methods for communicating 3D concepts. Hence, there is a need in the field of SHM for a method for visualizing sensor networks and data that enables 3D understanding, yet does not necessitate building a 3D digital model.

**Figure 2:**
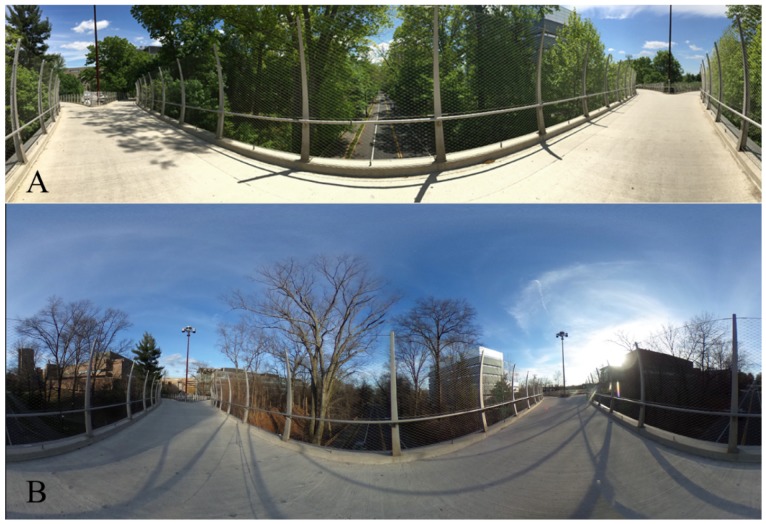
(**A**) Planar panorama (**B**) Spherical panorama (modified from [29]).

Virtual tours (VT) have been implemented for tourism and educational purposes to communicate 3D space to a broad audience in a simple and intuitive manner [27,28]. VT is an immersive environment made up of connected and interactive spherical panoramas. Spherical panoramas differ from planar panoramas in the amount of space they convey to a user. Planar panoramas, the more common type, illustrate a horizontal 360° view around the camera that does not include views of the floor or ceiling (Figure 2A).

Previously, scholars have suggested that a combination of Virtual Reality (VR) and Informational Modeling (IM) could document a building [30,31,32]. Informational modeling means that relevant data such as Acrobat Reader files (PDF-files), images, or databases in various formats can be included in the viewing environment [30]. A VT/IM-based method for SHM could fill the gap in methods for SHM sensor network and data visualization, identified in Figure 1. While this approach has been successfully applied to conservation of cultural heritage sites [29] and preliminarily tested for SHM [33], it has not yet been fully applied and validated for SHM purposes. In the next sections, a new method for SHM data visualization based on VT/IM is outlined and evaluated through application on Streicker Bridge on Princeton University’s campus, which is equipped with an existing SHM system.

## 3. Case Study: Streicker Bridge

Streicker Bridge is a pedestrian bridge on Princeton University’s campus. The bridge comprises a main span and four approachways referred to as “legs” throughout this paper. The main span is a deck-stiffened arch and the legs are continuous curved girders supported by steel columns. The arch and the columns are made of weathering steel while the deck of the main span and the legs is reinforced, post-tensioned concrete.

**Figure 3:**
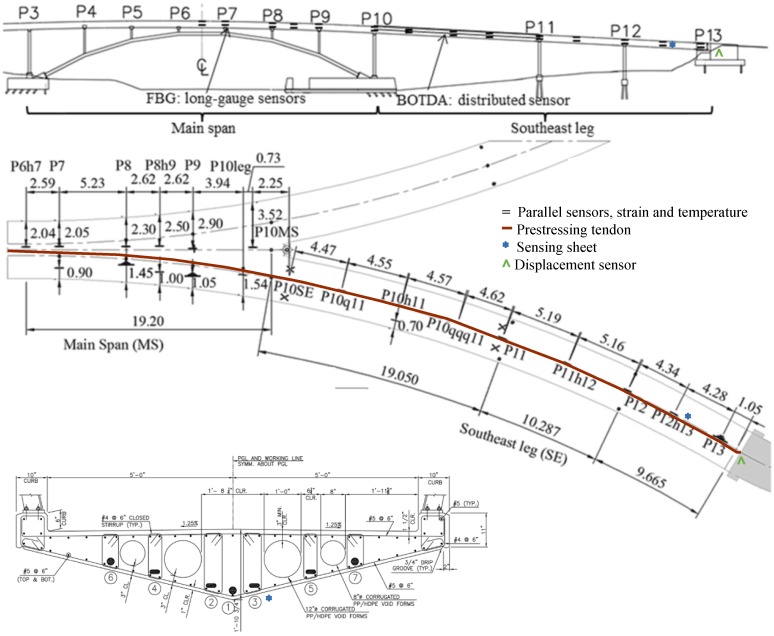
Locations of the strain and temperature sensors for Streicker Bridge. The cross-sectional view is at the location of the displacement sensor on the South-East leg [36].

This bridge was outfitted with two different fiber-optic sensing technologies: (1) discrete Fiber Bragg-Grating (FBG) long-gauge sensing technology and (2) truly distributed sensing technology based on Brillouin Time Domain Analysis [34]. Both approaches measure average strain and temperature at their locations on the bridge. Additionally, the bridge has FBG-based displacement sensors at the abutment of south-east leg [35], and a prototype of a sensing sheet—a novel two-dimensional quasi-distributed strain sensor based on large-area electronics [9]. The locations of the sensors can be seen in Figure 3.

**Figure 4:**
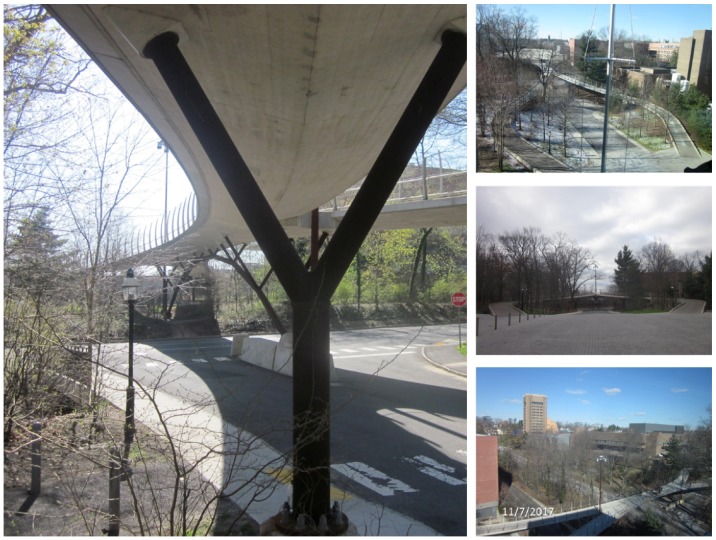
Photographs illustrating complex 3D geometry of Streicker Bridge [37].

Figure 3 depicts a side, plan, and cross-sectional view of Streicker Bridge. Illustrating the locations of sensors, the side-view shows the location of the different sensor typologies on the main span and the South-East leg including parallel strain and temperature sensors, a sensing sheet, and a displacement sensor. The plan view shows the locations of those sensors in addition to the location of the prestressing tendon. Lastly, the cross-section illustrates the same sensors as the main span with the addition of the sensing sheet. Due to the complex 3D shape of the bridge (Figure 4), and 3D topology of sensors, as well as the heterogenous typology and composition of the SHM system (Figure 3), Streicker Bridge is a good candidate for testing the new 3D data visualization method based on VT/IM method [37].

## 4. Tools and Software Used for VT/IM

For this project, a RICOH THETA S camera, 12 megapixels, was used to capture the spherical panoramas [38]. A total of 27 spherical panoramas were used to capture Streicker Bridge entirely. This number of panoramas enabled us to recreate the complete bridge virtually including views from the top and bottom. The RICOH THETA S camera automatically converts the images it takes to panoramas, which expedites the process of making the virtual tour. If a different camera is used to take the images, programs such as Hugin or PTGUI can be used to stitch the images into spherical panoramas [39,40]. The spherical panoramas were brought into Photoshop to remove the tripod from the field of view as well as correct any lighting discrepancies between adjacent panoramas. By removing the tripod from the panoramas, a user has an uninhibited experience of the bridge. This is particularly important for the images taken on top of the bridge deck as to not hinder the view of sensors embedded in the deck. With the tripod removed, the sensor location is more clearly conveyed.

The software program Kolor Panotour Pro was used to stitch the spherical panoramas together into the virtual tour environment [41]. A customized interface was created using this software that enables a user to access embedded and/or internet-/ethernet-accessible SHM data and metadata in various formats—databases, image galleries, texts, graphs, etc. (e.g., formatted as PDF, jpg, etc.), and other items through what are called called “hotspots”. A “hotspot” is an on-click conditional that allows a user to click on a certain part of the panorama, and a predefined event occurs. For example, if a user clicks on a strain sensor in the virtual environment, they can be brought to a database with the strain measurements. For this case study, the following was included in the VT/IM environment:

1. Metadata (structure)
  - Technical images showing sensor location in cross-sectional, aerial, and side views;
  - Diagram showing the post-tensioning profile of South-East Leg.
2. Metadata (SHM system)
  - Information box detailing the resolution, repeatability, typical gauge length, dynamic range, and maximum measured frequency of the strain sensors;
  - Legend showing various types of sensors;
  - Color-coding scheme that identifies function, malfunction, or disconnection of the sensors.
3. Data (raw)
  - Databases connected to the strain sensors showing the raw strain data over time;
  - Databases connected to the sensing sheet showing the raw strain data for each strain sensor over time;
  - Databases connected to the displacement sensors showing the raw displacement data over time.
4. Data (analyzed)
  - Graphs connected to the temperature sensors showing the relationship between temperature data and the time of day;
  - Diagrams showing curvature and displacement graphs for South-East Leg.
  - Diagram showing the pre-stressing force in South-East Leg.

These objects were integrated in this case study as per previous work that defined SHM data visualization principles [2]. Only some of the objects discussed in that paper were embedded to demonstrate feasibility and study the performance of the VT/IM method; the other objects could be integrated as well, but are considered outside the scope of this work.

## 5. Results and Discussion

The topologies of the Streicker Bridge and sensor network on Streicker Bridge are complex, and understanding the SHM system and SHM data requires 3D visualization and accessibility methods, as 2D representations are less intuitive and might create confusion. There was not an existing 3D model of the bridge and there was no interest expressed by those working on other (non-SHM) aspects of Streicker Bridge project to create one. Therefore, the VT/IM method was implemented, in attempt to fill the gap in the decision model shown in Figure 1.

### 5.1. Navigating the Interactive Interface

To enable a user to visualize a 3D sensor network and communicate with others working on a project, an interactive interface was developed and applied. A user can navigate the virtual tour environment in three main ways.

**Figure 5:**
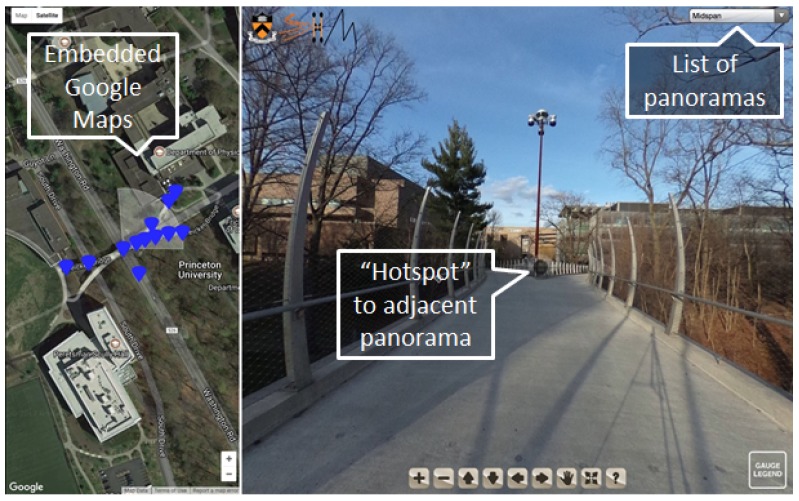
Virtual tour interface illustrating the different types of navigation: (1) embedded Google Maps, (2) “hotspot” connections to adjacent panoramas, and (3) drop-down menu with list of all panoramas of the bridge.

1. A user can interact with a built-in map, driven by Google maps; here a user can see the different viewpoints available, select one, and be transported virtually to this location on the bridge (see Figure 5);
2. A user can use built in “scene-connectors” to virtually “walk” from one view of the bridge to another; if a user is on one part of the bridge deck, they can move to an adjacent position along the deck by clicking on the appropriate “hotspot” in the virtual environment (see Figure 5);
3. Last, a user can select where to navigate to through a drop-down menu. This allows a user to navigate to a specific location without having to know where it is on a map. Figure 5 illustrates these means of navigating the VT/IM environment.

### 5.2. Access to and Visualization of SHM Data and Metadata

**Figure 6:**
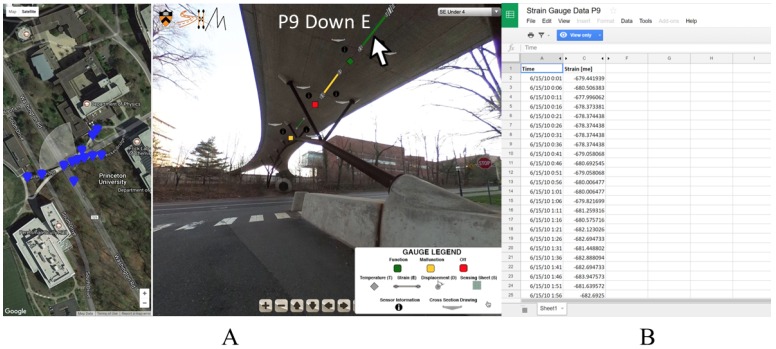
(**A**) When a user hovers over a sensor, they can see its ID as well as what it is measuring; (**B**) When a user clicks on the sensor they can access a database with time series.

**Figure 7:**
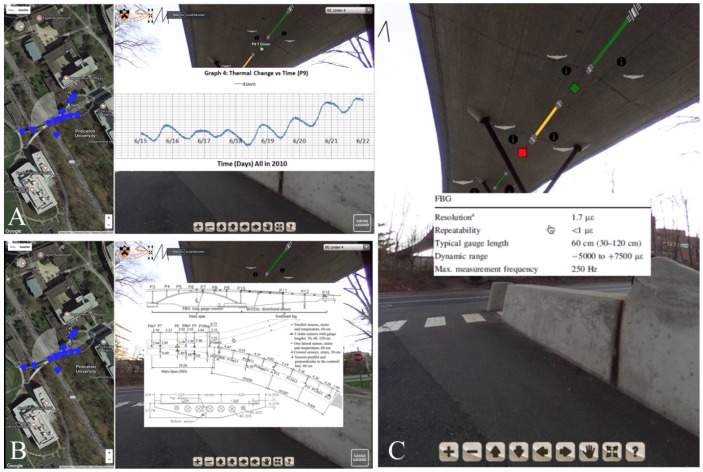
Examples of VT interactions showing (**A**) thermal change over time; (**B**) technical drawings, and (**C**) strain sensor metadata.

As discussed above, an advantage of using a VT/IM environment is interactive accessibility to information through “hotspots”. The example described earlier enables an SHM practitioner to click on a sensor in the virtual environment and be brought to a database of strain values. In the VT/IM environment, a user can access local, raw data (strain, temperature, displacement) and global, analyzed data (prestress force distribution, curvature distribution, deformed shape), and metadata relative to structure (technical drawings, prestressing scheme) or relative to SHM system (color coding of the sensors, specifications of the monitoring system). Some examples of how these hotspots were integrated into our virtual tour can be seen in Figure 6, which features hotspots for a temperature sensor and a strain sensor. The interactive legend in the bottom right corner of Figure 6A illustrates the type and the current state of each sensor (i.e., functioning, malfunctioning, disconnected). Figure 6B illustrates what happens when a user clicks on the sensor. Here, a user can access the database storing the time series of strain for this sensor and export it if desired. Examples of other objects that can be visualized such as thermal change over time, technical drawings, and strain sensor metadata can be seen in Figure 7.

While accessibility to this data is beneficial for practitioners of Structural Health Monitoring, it is imperative that the sensor data cannot be accessed or manipulated by parties not associated with the project. For this reason, this work used Panotour Pro for the generation of the virtual tours since protection features are built in at each part of the pipeline. As a first-layer of protection, the original XML files used to generate the virtual tour can be encrypted directly from the Panotour Pro software [41]. In addition to this, a user can chose to restrict the domains which are authorized to host the tour in order to control access [41]. Furthermore, if restricted domains are implemented, the virtual tour is compatible with protected websites. If this option is selected, the tour will only be displayed on a website where there is password protection on the server side [41].

### 5.3. Evaluation of VT/IM Performance

Two evaluation criteria were applied to assess the performance of the proposed visualization method. The first criterion assesses improvements in terms of ease of access to and visualization of SHM sensor networks, data, and metadata, and in particular targets comprehension of SHM system, its results, and their relationship to the monitored structure. The second criterion evaluates efficiency of the method as a 3D visualization tool, compared with the other visualization methods that would require building of 3D model, as per Figure 1.

**Figure 8:**
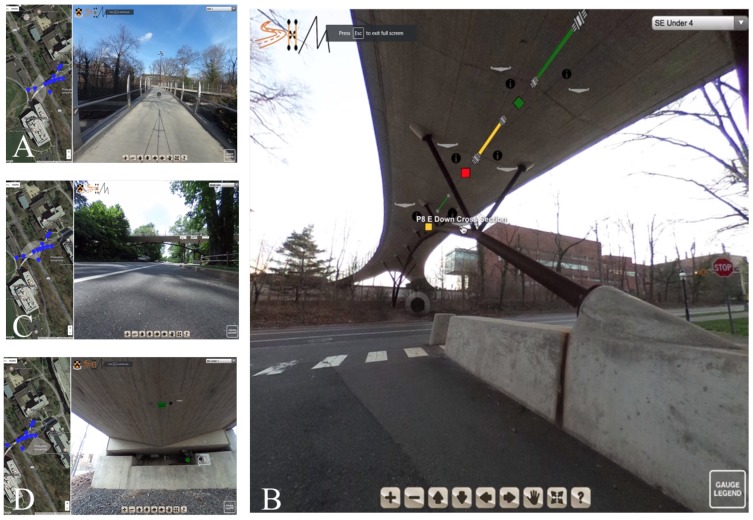
Stills of virtual (VT/IM) environment: (**A**) Scene 1 showing navigation elements; (**B**) Scene 2 showing sensors under the bridge; (**C**) Scene 3 showing global data on a side-view of the bridge; (**D**) Scene 4 showing displacement sensor and sensor sheet under South East leg.

In the VT/IM environment, a viewer can virtually walk around, under, and on top of the structure by the means of navigation illustrated in Figure 5. On this tour, a user can see “hotspots” that can bring them to the positions where sensors are located on the bridge. A user can interact with these sensors to get further information about the sensors: the sensor ID, as well as the raw and analyzed data in databases where the data collected from the sensor is stored. The main features of the method are presented in Figure 8A–D. However, to fully assess the performance of the method, a demonstration video showing a few different scenes of the bridge was prepared and can be found at the following link: https://vimeo.com/234006206.

1. The first scene (see Figure 8A) familiarizes the user with the interface and the virtual tour environment. There is a video of a user panning around the top of the bridge, viewing the different hotspots they can interact with (the black circles). Additionally, a user can see how the geographic map updates as the user navigates the space. The blue markers on the left-hand side illustrate to a user where navigation hotspots are and the white fan around that pin shows the current field of view to orient a user. In the first scene, a user interacts with two of the hotspots to navigate underneath the bridge.
2. The second scene (see Figure 8B) is set under the bridge and shows a user where sensors are on the bridge and what types of sensors are there. Here three strain sensors can be seen with three accompanying temperature sensors. For each of the sensors, there is an information hotspot detailing the resolution, repeatability, typical gauge length, dynamic range, and maximum measurement frequency. Additionally, since this information was available, there is a picture of the cross section included, showing where in the cross section the sensor is located. The colors of the sensors in the scene correlate to their current function as indicated by the gauge legend in the lower right-hand corner: functioning, malfunctioning, disconnected. The legend describes to a user what the different sensors are in the tour (temperature sensor, Fiber Bragg-Grating discrete long-gauge, displacement, and a sensing sheet), what their icons look like, and what their colors indicate. If a user hovers over the sensor, they can get its name. By clicking on it, a user can interact with the data as an image or data file.
3. The third scene is under the South-East leg of the bridge and displays a sensing sheet and displacement sensor (see Figure 8C). Again, a user can access the information from this sensor in either an embedded image or linked data file.
4. The fourth scene is on the side of the bridge and provides a side view. Global, analyzed data such as curvature and displacement diagrams of the South-East leg, the post-tensioning tendon profile, and the prestressing force in the South-East leg are integrated into this scene (see Figure 8D).

To assess improvements in terms of ease of access to and visualization of SHM sensor networks, data, and metadata, a short survey was conducted among graduate and undergraduate students at Princeton University. This method of evaluation has been utilized successfully in previous work to assess the performance of SHM visualization programs [2]. While students understood basic civil engineering principles and participated in course on SHM, they lacked real-life experience in SHM and were unfamiliar with Streicker Bridge project. All this combined made them appropriate audience to evaluate the VT/IM environment. The three-minute-long video linked above and an accompanying short survey were sent to the students, so the students could watch the video and write their feedback.

A short description outlining the aims of the project (107 words) was given at the beginning of the survey, but the SHM system presented in the video was not described. The survey comprised the following questions:

1. How easy was it to understand what the video shows?
2. Does the video help to understand the SHM system and sensor network installed on the bridge?
3. Does the video help assess the behavior/ functionality of the sensors on the bridge?

Each of the above-mentioned questions was scored on a linear scale from 1 to 5 where 1 indicated that the student did not understand and 5 indicated that the student completely understood. In addition to the numeric value assigned, the students were required to provide short paragraphs clarifying their answers. Furthermore, the students were asked if they had any other comments (positive or negative) about the video and if they had suggestions for improvements. The list of questions was brief to encourage student participation and they included both a numeric value to aid in quantification and open-ended description to catalyze critical thinking.

Akin to a similar study [2], the validation criteria was set to 50% of positive feedback on questions 1–3 where a positive value is scored as a 4 or 5, a neutral value is scored as a 3, and a negative value is scored as a 1 or 2. This criterion accounts for both the inexperience of SHM students and lack of information given on the project.

**Figure 9:**
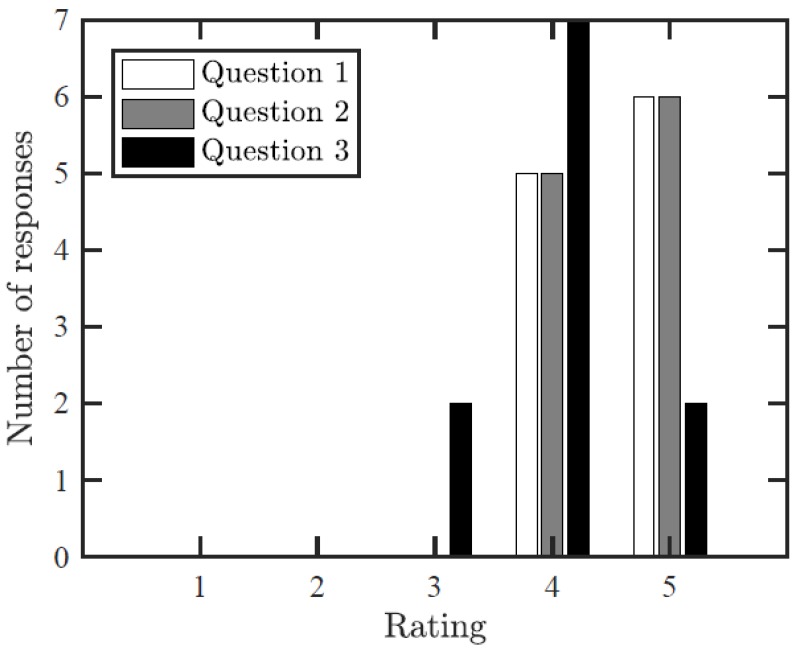
Bar chart reflecting answers to survey questions. The scale for each question was from 1–5 with 1 being the lowest.

Eleven students in total responded to the survey to validate the VT/IM environment. A graph of the responses can be seen in Figure 9. Figure 9 indicates that all students found the VT/IM environment to be easy to understand. In examining the open-ended answers to the survey questions, it seems that only confusion about the video came when the user was taken below the bridge. It was indicated that if a different looking hotspot was used, one that indicated downward motion, that might have made it clearer to the viewer. Like the first question, in the second part of the survey all students claimed to understand the SHM system and sensor network installed on the bridge without prior knowledge and in the open-ended section it was remarked that this system helped the users to gain perspective about how the sensors were related to the bridge and to each other. Lastly, 9 students (81%) found the virtual tour useful for assessing the behavior/functionality of the sensors on the bridge (Question 3). The users found that the system provided good information about sensor typology and location, but more hotspots discussing the global behavior of the bridge should be added. This is something that future generators of virtual tours should consider. In the sections for “Other comments” and “Suggestions for improvements”, it was stated that the ability to move from between different viewpoints on the bridge was crucial to understanding the overall structure of the SHM system while it was somewhat overwhelming as a user since there was a lot to take in with each scene. This example was to show the functionality of the tool; future users should take care to optimize the amount of information in a scene to minimize any overwhelming effects.

**Figure 10:**
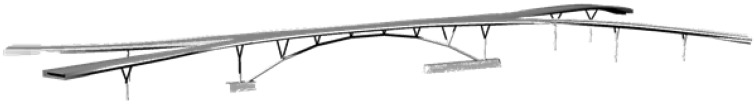
Perspective rendering of the 3D model for Streicker Bridge.

While a 3D model was not required by other parties working with Streicker Bridge, for direct comparison of efficiency of proposed method, in this study a crude 3D model of the bridge was created in addition to the VT/IM environment. This model can be seen in Figure 10. Table 1 is a comparison of the completion time and data storage required for each approach.

Considering the differences in terms of time and data savings, for projects where a 3D understanding of the structure is necessary, but there is no future use of a 3D model (e.g., in future structural analysis, education purposes, etc.), a 3D model is inefficient. Concordant results were obtained in another study that compared VT/IM environment vs. 3D methods of documenting a structure such as laser scanning and photogrammetry. Finding VT/IM to be 16 times faster than laser scanning and 1/30 the cost, this previous study illustrated the efficiency of VT/IM environments for conservation of cultural heritage sites [29]. Thus, under the circumstances where a 3D model is outside the broader project’s scope, VT/IM is an efficient method for accessing and visualizing SHM sensor networks, data, and metadata.

## 6. Conclusions

This work identified the current gap in methods for accessing and SHM data visualization, in particular when the topological complexity of an SHM system and monitored structure calls for 3D visualization, but creating 3D model is out of the broader scope of the project. It was found that the method proposed in this work, which is based on VT/IM, could be an efficient means of addressing the above challenge. In this method, a user can first document their structure using spherical panoramas and connect adjacent views to ease 3D understanding of the structure. To augment the communication process, images, informational text, and data files can be directly linked to the environment and accessed by the user. This enables a user to quickly familiarize themselves with the structure and the SHM system, understand where the data is coming from on the structure, and see how results of data analysis relate to the structure. This in turn can help them identify and diagnose unusual behaviors. Effectiveness and efficiency of VT/IM were successfully tested on Streicker Bridge, and evaluated through a survey and comparison with roughly made 3D model. The VT/IM method opens new doors and transforms current practices in SHM data visualization which is vital to the overall process of monitoring.

## Figures and Tables

**Table 1:** Direct comparison of the completion time and data storage requirements for VT/IM and 3D modeling in case of the Streicker Bridge.

Method	Time Spent (h)	Data File Size (MB)
VT/IM	1	87.9
3D modeling	12	4000
